# CRUSE – What the first 100 days have taught us

**DOI:** 10.5339/qmj.2023.sqac.14

**Published:** 2023-11-19

**Authors:** Sophia Neisinger, Bernardo Sousa Pinto, Aiste Ramanauskaite, Jean Bousquet, Karsten Weller, Martin Metz, Markus Magerl, Emek Kocatürk, Ivan Cherrez Ojeda, Ana M Gimenez-Arnau, Marcus Maurer

**Affiliations:** ^1^Institute of Allergology, Charité – Universitätsmedizin Berlin, Corporate member of Freie Universität Berlin and Humboldt-Universität zu Berlin, Berlin, Germany Email: sophia.neisinger@charite.de; ^2^Fraunhofer Institute for Translational Medicine and Pharmacology ITMP, Allergology and Immunology, Berlin, Germany; ^3^Department of Dermatology, Urticaria Center of Reference, and Excellence (UCARE), Koç University School of Medicine, Istanbul, Turkey; ^4^MEDCIDS-Department of Community Medicine, Information and Health Decision Sciences, Faculty of Medicine, University of Porto, Porto, Portugal; ^5^CINTESIS-Center for Health Technology and Services Research, University of Porto, Porto, Portugal; ^6^RISE-Health Research Network, University of Porto, Porto, Portugal; ^7^Universidad Espíritu Santo, Samborondón, Ecuador, Respiralab Research Center, Guayaquil, Ecuador; ^8^Department of Dermatology, Urticaria Center of Reference, and Excellence (UCARE), Hospital del Mar, IMIM, Universitat Pompeu Fabra, Barcelona, Spain

## Abstract

**Introduction:** Health apps play an increasing role in everyday healthcare, especially for chronic diseases. The Chronic Urticaria Self Evaluation (CRUSE) is a new mobile health app for chronic spontaneous urticaria (CSU) patients, which replaces disease tracking via paper and pen, thus making disease monitoring more convenient, increasing tracking compliance, and improving data quality and access.

**Methods:** CRUSE enables patients to complete patient-reported outcome measures on their smartphone and send the results, along with current medication and pictures, to their treating physician via email. CRUSE captures the urticaria (UAS) and angioedema activity (AAS) scores and the urticaria and angioedema control tests (UCT and AECT). In this work, a descriptive analysis of CRUSE users and reported days was performed. The global network of Urticaria Centers of Reference and Excellence (UCARE) provides the app and its data.

**Results:** CRUSE is now available in Germany, Switzerland, Austria, the UK, Italy, Spain, France, and Turkey. Of 620 newly registered users (from July 1^st^ until November 18^th^ of 2022), 72 % were female, and the mean age was 36.6 years (17 - 78 years). The average daily UAS and AAS value (mean ± standard deviation) were 2.1 ± 1.9 and 7.2 ± 3.3, respectively. Most CRUSE patients had poorly controlled disease, with mean UCT values of 7.0 ± 4.4 and mean AECT values of 8.1 ± 4.5.

**Conclusion:** The first days of patients with CSU using CRUSE confirm the high need for an app that helps to monitor disease activity, impact, and control. The first results indicate low levels of disease control in most CRUSE users, with low UCT and AECT values. Future analyses will assess follow-up documentation data and evaluate the effects of treatment changes on CSU activity, impact, and control.

